# Ontogeny of Hepatic Energy Metabolism Genes in Mice as Revealed by RNA-Sequencing

**DOI:** 10.1371/journal.pone.0104560

**Published:** 2014-08-07

**Authors:** Helen J. Renaud, Yue Julia Cui, Hong Lu, Xiao-bo Zhong, Curtis D. Klaassen

**Affiliations:** 1 Department of Internal Medicine, University of Kansas Medical Center, Kansas City, Kansas, United States of America; 2 Department of Pharmacology, SUNY Upstate Medical University, Syracuse, New York, United States of America; 3 Department of Pharmaceutical Sciences, University of Connecticut School of Pharmacy, Storrs, Connecticut, United States of America; 4 College of Medicine, University of Kansas, Kansas City, Kansas, United States of America; Northeast Ohio Medical University, United States of America

## Abstract

The liver plays a central role in metabolic homeostasis by coordinating synthesis, storage, breakdown, and redistribution of nutrients. Hepatic energy metabolism is dynamically regulated throughout different life stages due to different demands for energy during growth and development. However, changes in gene expression patterns throughout ontogeny for factors important in hepatic energy metabolism are not well understood. We performed detailed transcript analysis of energy metabolism genes during various stages of liver development in mice. Livers from male C57BL/6J mice were collected at twelve ages, including perinatal and postnatal time points (n = 3/age). The mRNA was quantified by RNA-Sequencing, with transcript abundance estimated by Cufflinks. One thousand sixty energy metabolism genes were examined; 794 were above detection, of which 627 were significantly changed during at least one developmental age compared to adult liver. Two-way hierarchical clustering revealed three major clusters dependent on age: GD17.5–Day 5 (perinatal-enriched), Day 10–Day 20 (pre-weaning-enriched), and Day 25–Day 60 (adolescence/adulthood-enriched). Clustering analysis of cumulative mRNA expression values for individual pathways of energy metabolism revealed three patterns of enrichment: glycolysis, ketogenesis, and glycogenesis were all perinatally-enriched; glycogenolysis was the only pathway enriched during pre-weaning ages; whereas lipid droplet metabolism, cholesterol and bile acid metabolism, gluconeogenesis, and lipid metabolism were all enriched in adolescence/adulthood. This study reveals novel findings such as the divergent expression of the fatty acid β-oxidation enzymes Acyl-CoA oxidase 1 and Carnitine palmitoyltransferase 1a, indicating a switch from mitochondrial to peroxisomal β-oxidation after weaning; as well as the dynamic ontogeny of genes implicated in obesity such as Stearoyl-CoA desaturase 1 and Elongation of very long chain fatty acids-like 3. These data shed new light on the ontogeny of homeostatic regulation of hepatic energy metabolism, which could ultimately provide new therapeutic targets for metabolic diseases.

## Introduction

The liver plays a central role in sustaining metabolic homeostasis by maintaining a constant supply of energy fuels to bodily tissues. It is the critical relay point for the reception of energy substrates arising from food digestion or degradation of endogenous sources, their metabolic conversion or storage, and the final redistribution to bodily tissues. In this regard, the liver uses carbohydrates, free fatty acids, and amino acids to generate and export two principal energy substrates, glucose and ketone bodies, that can be used for energy generation by other tissues. Additionally, the liver produces very-low density lipoprotein (VLDL) particles to transport triglycerides to adipose tissue for storage. Consequently, as the predominant inter-conversion point for energy substrates in mammals, the liver plays an essential role in the adaptive metabolic response during daily fasting-feeding cycles as well as long-term changes in nutrition [1], [2], [3].

During fasting or following exercise, hormones cue the liver to maintain blood glucose levels through two processes: gluconeogenesis (generation of glucose from non-carbohydrate carbon substrates such as glycerol, lactate, and amino acids); and glycogenolysis (degradation of glycogen). During prolonged fasting or starvation, the liver breaks down fatty acids through a process known as ketogenesis to produce ketone bodies that can be used by most extrahepatic tissues as an energy substrate. These processes are inhibited following ingestion of a meal, concomitant with stimulation of hepatic glycogen synthesis (glycogenesis), primarily in response to high circulating insulin levels [reviewed in [4]]. In addition, insulin stimulates hepatic lipase, which facilitates the uptake of free fatty acids (FFA) derived from gut chylomicron remnants into the liver. FFAs are subsequently esterified into triglycerides, and then packaged into VLDL particles that are exported and stored in other tissues as an additional energy substrate.

During growth and development there are fluctuating demands for energy substrates as well as dramatic changes in nutrition, as offspring transition from fetus to postnatal life, and from pre- to post-weaning. *In utero*, the main energy substrate transferred across the placenta is glucose [5]. However, after birth there is a sudden change in energy substrate availability due to the consumption of high-fat, low-carbohydrate milk. Thus, a metabolic adaptation at birth is necessary to maintain blood glucose levels. In rodents, this adaptation is accomplished by activation of gluconeogenic, lipid oxidative, and ketogenic pathways [6]. The suckling-weaning transition is also accompanied by a profound change in nutrition. As weaning approaches, milk intake is gradually replaced by intake of higher-carbohydrate, lower-fat, solid foods [6]. To ensure survival, neonates must successfully adapt to these changes in nutrition throughout development, which requires significant changes in energy substrate metabolism. Thus, hepatic energy metabolism is dynamically regulated throughout different life stages. However, mechanisms governing this regulation are not completely understood.

The purpose of this study is to provide a comprehensive quantification of the mRNA abundance of energy metabolism genes during liver development. To accomplish this, we used RNA-Sequencing, which has the distinct advantage of enabling us to determine the true quantification of transcripts, and is not delimited by the requirement for primers or probes as is the case with other mRNA detection tools such as Northern blotting, PCR, or DNA microarray. Using mouse ontogenic development as a tool, we hope to gain a better understanding of the developmental regulation of energy metabolism genes, which may lead to novel therapeutic targets for pathologies associated with aberrant metabolic gene regulation.

## Methods and Materials

### Ethics Statement

The animal housing facility at the University of Kansas Medical Center is accredited by the Association for Assessment and Accreditation of Laboratory Animal Care. All procedures were approved by the University of Kansas Medical Center's Institutional Animal Care and Use Committee.

### Animals

Eight-week-old C57BL/6J mice were purchased from Jackson Laboratories (Bar Harbor, ME). Mice were housed in a temperature-, light-, and humidity-controlled environment. All animals were given *ad libitum* access to water and standard rodent chow (Harlan Teklad 8604; Halan Teklad, Madison, WI). Mice were bred overnight and separated the next morning. Pups that were used for ages after weaning were weaned at 21 days of age. All mice were euthanized by a pentobarbital overdose. Livers from offspring were collected at the following 12 ages: gestational day 17.5 (GD17.5), day 0 (immediately after birth and before the start of suckling), day 1 (exactly 24 h after birth), as well as days 3, 5, 10, 15, 20, 25, 30, 45, and 60 (collected at 9:00 a.m.). Due to potential variations caused by the estrous cycle in maturing adult female mice, only male livers were used for this study (*n* = 3 per age, randomly selected from multiple litters). Isolated livers were frozen immediately in liquid nitrogen and stored at −80°C.

### RNA isolation

Total RNA was isolated from liver tissue using RNA-Bee RNA Isolation Reagent (Tel-Test Inc., Friendswood, TX) according to the manufacturer's protocol. RNA concentrations were quantified using a NanoDrop Spectrophotometer (NanoDrop Technologies, Wilmington, DE) at a wavelength of 260 nm. RNA Integrity was evaluated using an Agilent 2100 Bioanalyzer (Agilent Technologies Inc., Santa Clara, CA) at the University of Kansas Medical Center Genome Sequencing Facility. Samples with RNA integrity values above 7.0 were used for the following experiments.

### Complementary DNA library preparation

The cDNA libraries were prepared from total RNA by an Illumina TruSeq RNA sample prep kit (Illumina, San Diego, CA). According to the recommendations of the manufacturer, 3 µg of RNA were used as the RNA input. Poly-T primers were used to select the poly-A containing mRNAs from RNAs. According to the manufacturer's protocol, the RNA fragmentation, first and second strand cDNA syntheses, end repair, adaptor ligation, and PCR amplification were performed (Illumina, San Diego, CA). Excluding adapters, the average cDNA library size was approximately 160 bp. The cDNA libraries were evaluated for RNA integrity and quantity using an Agilent 2100 Bioanalyzer (Agilent Technologies Inc., Santa Clara, CA).

### RNA-sequencing

Using a TruSeq 200 cycle SBS kit (Illumina), the cDNA libraries were clustered onto a TruSeq paired-end flow cell and sequenced for 100 bp paired-end reads (2×100) using an Illumina HiSeq2000 sequencer (University of Kansas Medical Center Genome Sequencing Facility). To ensure the data generated for each run were accurately calibrated during the image and data analysis, a phi X 174 (PhiX) bacteriophage genome as well as a universal human reference RNA sample were sequenced in parallel with other samples. In addition, the PhiX was spiked into each cDNA sample at approximately 1% as an internal quality control.

### RNA-Sequencing data analysis

Illumina's Real Time Analysis software was used for the pixel-level raw data collection, image analysis, and base calling. This software generated the base call files (*.BCL), which were converted to qseq files by the Illumina's BCL Converter. The qseq files were subsequently converted to FASTQ files for further downstream analysis. Using the mouse reference genome (NCBI37/mm9), the RNA-Seq reads from the FASTQ files were mapped to the mouse genome and splice junctions were identified by TopHat. The output files, which were in BAM (binary alignment/map) format, were analyzed by Cufflinks to estimate the transcript abundances. Cufflinks generated transcript structure predictions, which were then compared to the reference annotation Ensembl GTF version 65 by Cuffcompare. The mRNA abundances were expressed in FPKM (fragments per kilobase of exon per million reads mapped). RNA-Sequencing data was uploaded to the Gene Expression Omnibus database, accession number GSE58827.

### Western blot analysis

Protein expression of Scd1 was examined utilizing Western blotting. Liver tissue (approximately 50 mg) was homogenized in 1 ml T-PER Tissue Protein Extraction Reagent (Thermo Scientific, Rockford, IL)+10 µl Protease Inhibitor Cocktail (Sigma, St. Louis, MO). Protein concentration was quantified spectrophotometrically using the BCA assay (Biorad, Hercules, CA). Protein (20 µg) was loaded into 12% acrylamide-containing SDS-PAGE gels, and transferred to PVDF membranes. Membranes were then probed with antibodies specific for Scd1 (1∶1000 dilution; 2438S; Cell Signaling Technology, Inc., Danvers, MA) or α-tubulin (1∶5000 dilution; CP06; EMD-Millipore Corp, Billerica, MA). Following a one-hour incubation with species appropriate horse radish peroxidase-conjugated antibodies, membranes were immersed in Luminata Classico enhanced chemiluminescence reagent (EMD-Millipore Corp) followed by detection using GeneMate basic blue autoradiography film (Bioexpress, Kaysville, UT).

### Data visualization and statistics

A list of energy metabolism genes was compiled using the Gene Ontology (GO) database and the KEGG database, resulting in 1060 genes. The data for these energy metabolism genes were retrieved from the Cufflinks output for further analysis. Expression above background during at least one age of liver development was determined using the drop-in-deviance *F* test of the fitted FPKM data to a generalized linear model with a poison link function, a statistic designed to measure the significance of a gene's measured FPKM relative to a zero FPKM value. The *p* values were adjusted for extra Poisson variation and corrected for false discovery by the Benjamini-Hochberg method (Benjamini-Hochberg–adjusted false discovery rate [FDR-BH]) with a threshold of 0.05. To determine statistically significant changes in gene expression between ages, a one-way ANOVA was conducted followed by a Dunnett t (2-sided) post-hoc test using Day 60 as the control category. The ANOVA was carried out using SPSS v.20 software. The FPKM values were log2 transformed to achieve normal distribution prior to the ANOVA. Statistically significant differences were determined if p<0.05. One-way hierarchical clustering dendrograms were generated using the heatmap.2 function of the gplots package in R Bioconductor.

## Results

### Expression of genes involved in energy metabolism at different life stages

An average of 175 million reads were generated per sample by RNA-Seq and more than 80% of these reads were mapped to the mouse genome using TopHat. The mRNA expressions of 1060 genes that encode factors with known roles in energy metabolism were determined in livers of mice at twelve different ages. The selected list of 1060 genes was compiled from GO:0006629 (lipid metabolic process), GO:0006633 (fatty acid biosynthetic process), GO:0019395 (Fatty Acid Oxidation), GO:0009062 (Fatty Acid Catabolic Process), GO:0008203 (cholesterol metabolic process), GO:0008206 (bile acid metabolic process), KEGG synthesis and degradation of ketone bodies, lipid droplet and VLDL metabolism (compiled from the literature), GO:0006094 (gluconeogenesis), GO:0006096 (glycolysis), GO:0005980 (glycogen catabolic process), and GO:0005978 (glycogen biosynthetic process). Of these 1060 genes, 794 were significantly expressed during at least one age in liver (Benjamini-Hochberg-adjusted drop-in-deviance F test [FDR-BH], 0.05 in at least one of the twelve ages). Of these 794 genes, the expressions of 627 were significantly different in at least one age compared to Day 60 (adult).

**Figure 1:**
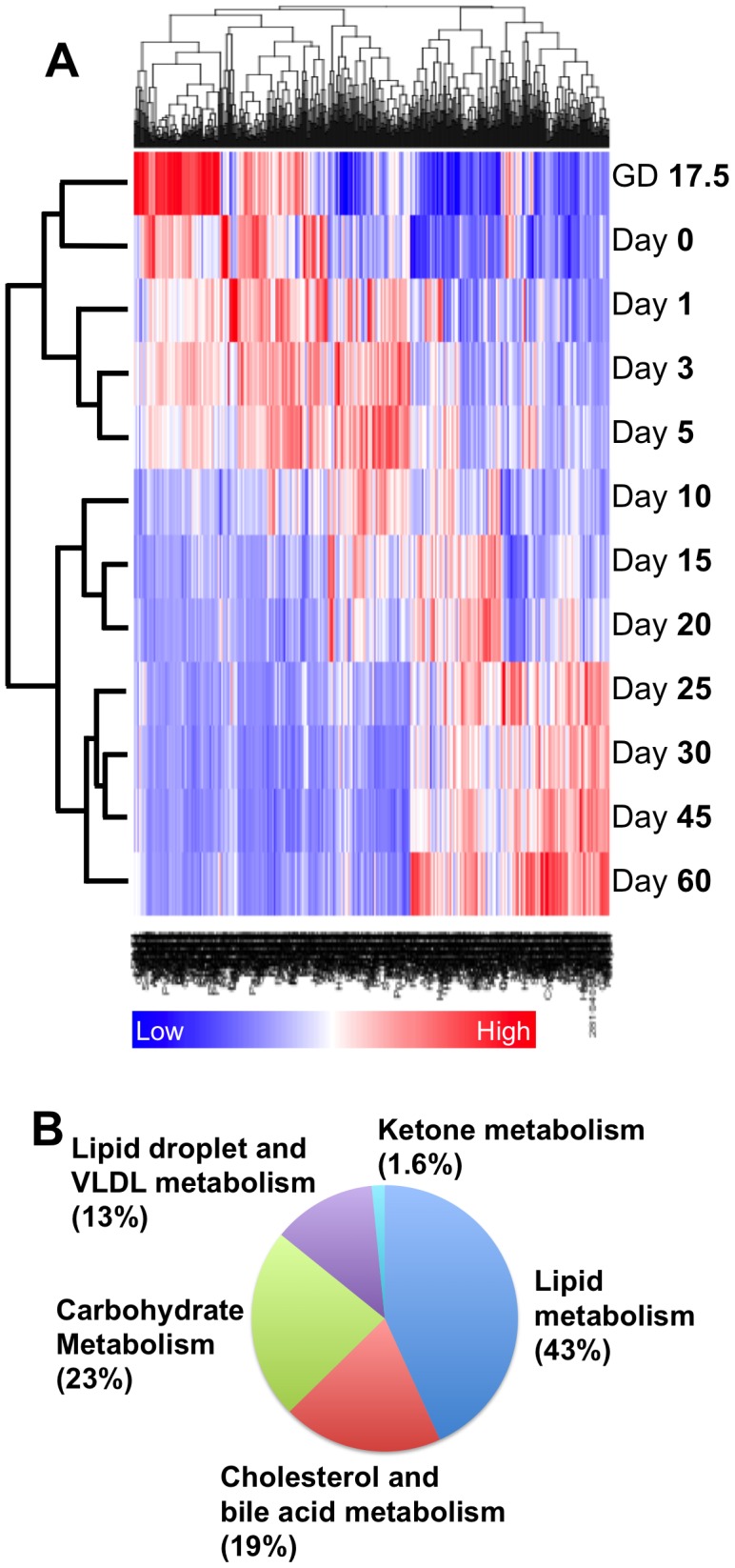
Ontogeny of energy metabolism gene expressions. A) Two-way hierarchical clustering of the 627 energy metabolism genes whose expression significantly changed in at least one age compared to Day 60. Genes clustered by age into 3 groups: perinatally-enriched (GD 17.5–Day 5), prepuberty-enriched (Day 10–20), and adolescent/adulthood enriched (Day 25–60). GD = gestational day. B) Pie chart of the percentages of the 627 energy metabolism mRNAs whose expression significantly changed in at least one age compared to Day 60 in each category of energy metabolism. VLDL = very low density lipoprotein receptor.

Two-way hierarchical clustering of the 627 genes whose expression significantly changed in at least one age compared to Day 60, showed that certain ages clustered together, namely GD17.5, Day 0, 1, 3 and 5 (perinatal-enriched); Day 10, 15, and 20 (pre-weaning-enriched); and Day 25, 30, 45, and 60 (adolescence/adulthood-enriched) (**Figure 1A**). The significant gene changes were in the following categories of energy metabolism: fatty acid metabolism (271 genes), cholesterol and bile acid metabolism (122 genes), carbohydrate metabolism (145 genes), lipid droplet and VLDL metabolism (79 genes), and ketone body metabolism (10 genes) (**Figure 1B**).

**Figure 2:**
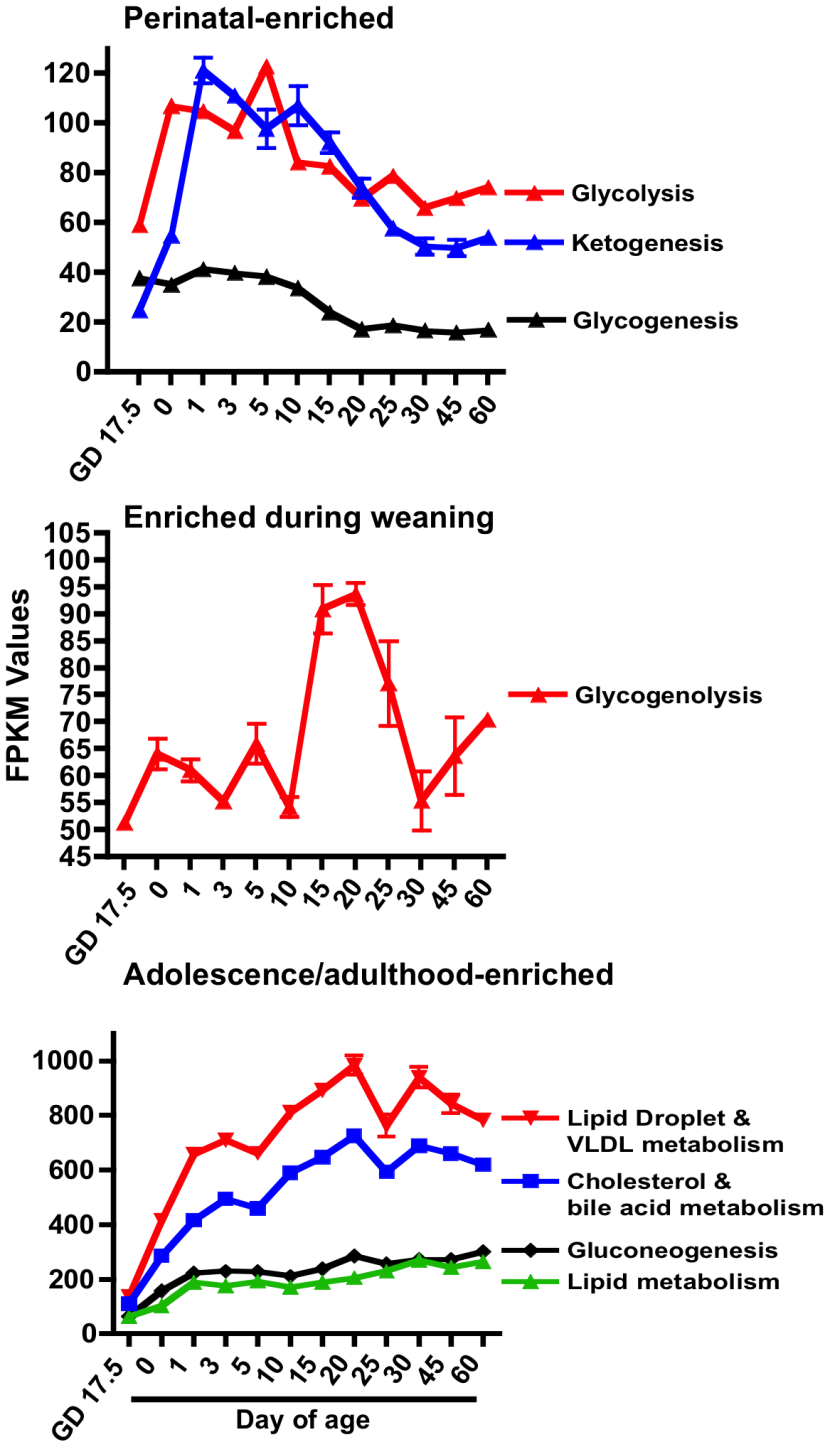
Ontogeny of the cumulative FPKM (fragments per kilobase of exon per million reads mapped) values for pathways of energy metabolism. Based on clustering analysis, energy metabolism pathways were clustered into adolescence/adulthood enriched pathways (top panel), prepuberty-enriched pathways (middle panel), and perinatally enriched pathways (bottom panel).

Clustering analysis of cumulative mRNA expression values for individual pathways of energy metabolism revealed 3 patterns of enrichment (**Figure 2**): glycolysis, ketogenesis, and glycogenesis were all enriched in the early postnatal period; glycogenolysis was the only pathway enriched around the weaning age; and lipid droplet and VLDL metabolism, gluconeogenesis, and lipid metabolism (including lipid synthesis and oxidation pathways) were all enriched in adolescence/adulthood. These results demonstrate that liver energy metabolism pathways dynamically change throughout development.

### Ontogeny of transcription factors that regulate energy metabolism

**Figure 3:**
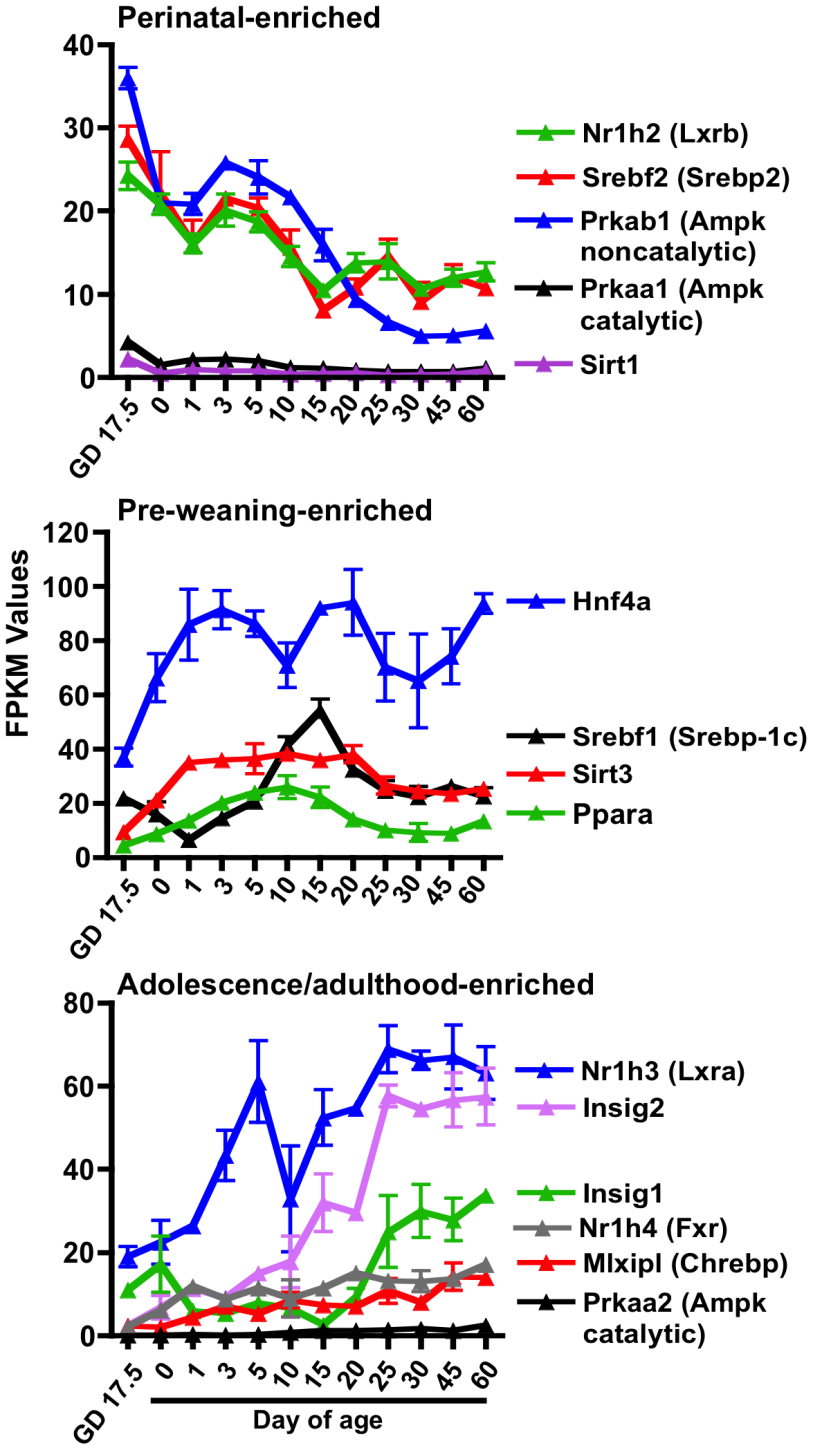
Ontogeny of the mRNA expression of known transcription factors that regulate intermediary metabolism. Based on clustering analysis, these transcription factors clustered into either adolescence/adulthood enriched (top panel), prepuberty-enriched (middle panel), and perinatally enriched (bottom panel).

A list of 22 transcription factors involved in energy metabolism was compiled from the literature. Nineteen of these genes were significantly expressed in at least one age. Of these 19 genes, the mRNA expressions of 14 genes were significantly different in at least one age compared to Day 60. Clustering analysis (not shown) grouped the transcription factors into three age-dependent clusters. Perinatal-enriched genes included LXRβ (Nr1h2), Srebp2 (Srebf2), Prkab1 (an Ampk noncatalytic subunit), Prkaa1 (an Ampk catalytic subunit), and Sirt1. Pre-weaning-enriched genes included Hnf4α, Srebp-1c (Srebf1), Ppara, and Sirt3. Whereas, LXRα (Nr1h3), Insig2, Insig1, Fxr (Nr1h4), ChREBP (Mlxipl), and Prkaa2 (an Ampk catalytic subunit) were all enriched in adolescence/adulthood (**Figure 3**).

### Expression of genes involved in lipid metabolism at different life stages

**Figure 4:**
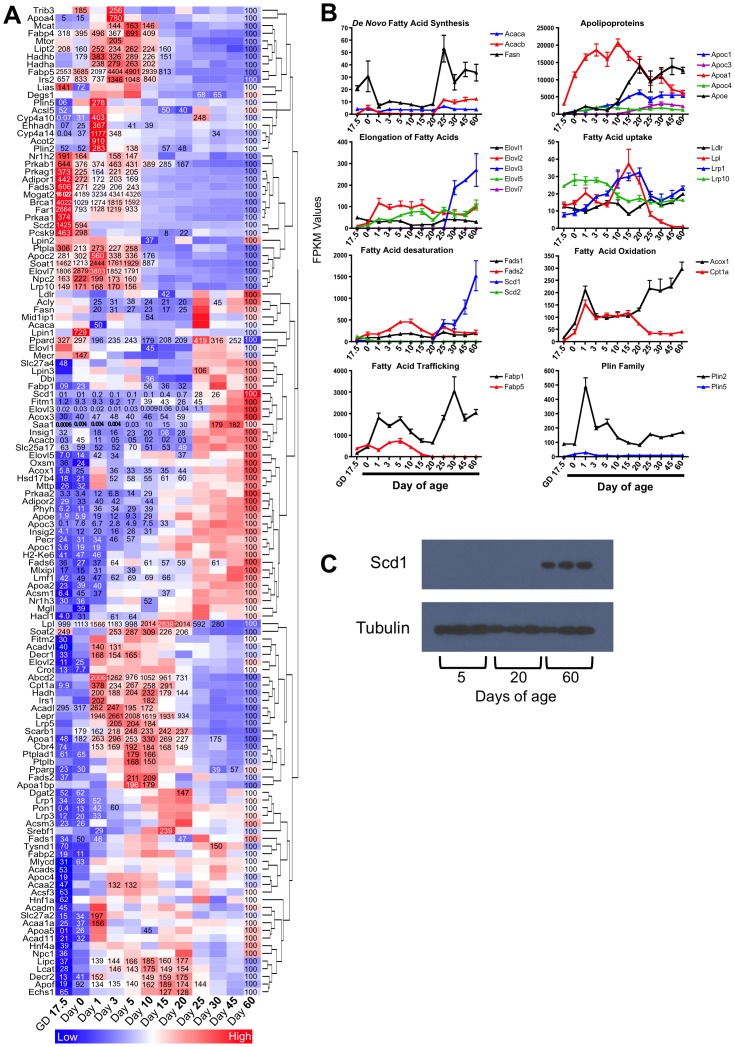
Ontogeny of lipid metabolism gene expressions. A) One-way hierarchical cluster map of lipid metabolism genes that changed significantly in at least one age compared to Day 60. mRNA expression of genes that were statistically different from those in mice 60 days of age (control) have the percent indicated within the heatmap square (control = 100%). B) Examples of FPKM (fragments per kilobase of exon per million reads mapped) values of individual genes of lipid metabolism throughout development. C) Western blot analysis of Scd1 protein expression in liver of pre-weaning and post-weaning mice, α-tubulin was used as a loading control. For gene function information see Table S1.

To evaluate the expression of genes involved in lipid metabolism, a list of genes was compiled using the Gene Ontology gene set GO:0006633 (Fatty Acid Biosynthetic process), GO:0019395 (Fatty Acid Oxidation), GO:0009062 (Fatty Acid Catabolic Process), and genes involved in lipid droplet and VLDL formation (compiled from the literature). The resulting list contained 256 genes. Of these 256 genes, 182 were significantly changed in at least one age compared to Day 60. This list was then filtered to eliminate genes exclusively involved in prostaglandin synthesis, leukotriene synthesis, sphingolipid metabolism, glycerolipid metabolism, oxidative phosphorylation, peroxisomal biogenesis, glyoxylate metabolism, antigen presentation, ethanol metabolism, and genes with unknown function; resulting in 129 genes. A one-way hierarchical cluster map of these 129 genes is displayed in **Figure 4A**. The FPKM values for genes specifically involved in de novo fatty acid synthesis, apolipoproteins, elongation of fatty acids, fatty acid uptake, fatty acid desaturation, fatty acid oxidation, fatty acid trafficking, and the perilipin (Plin) family genes at different ages are displayed in **Figure 4B**. Most notably, fatty acid synthase (Fasn) expression dramatically declined during the suckling stage of development (Day 1- weaning). Apolipoprotein A1 (Apoa1) expression was highest during the suckling period. The expressions of the elongase Elovl3, and stearoyl-CoA desaturase-1 (Scd1) were both robustly increased immediately after weaning. The expression pattern of Scd1 was confirmed at the protein level by Western blot analysis (**Figure 4C**). Acyl-CoA oxidase 1 (Acox1) and Carnitine palmitoyltransferase 1a (Cpt1a) (genes involved in peroxisomal and mitochondrial beta oxidation, respectively) showed a divergent expression pattern with Acox1 mRNA increasing and Cpt1a mRNA decreasing immediately after weaning.

### Expression of genes involved in ketogenesis at different life stages

**Figure 5:**
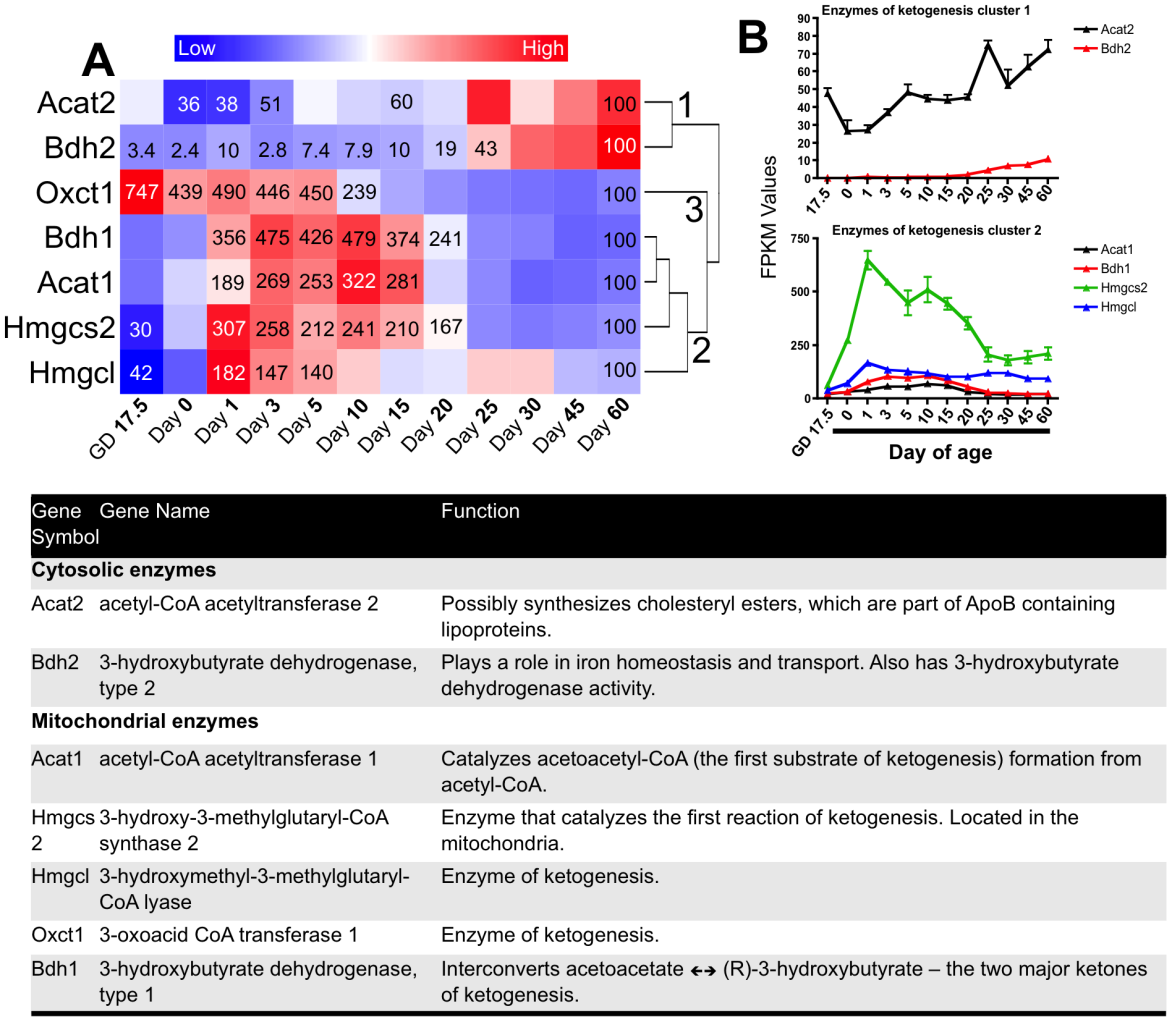
Ontogeny of ketone metabolism gene expressions. A) One-way hierarchical cluster map of ketone metabolism genes that changed significantly in at least one age compared to Day 60. mRNA expression of genes that were statistically different from those in mice 60 days of age (control) have the percent indicated within the heatmap square (control = 100%). B) Examples of FPKM (fragments per kilobase of exon per million reads mapped) values of individual genes of ketone metabolism throughout development.

To evaluate individual genes involved in ketogenesis, we used the list of genes from the KEGG Synthesis and Degradation of Ketone bodies gene set. This gene set included 10 genes, of which 7 were significantly changed compared to Day 60 in at least one age. A one-way hierarchical cluster map of these 7 genes is displayed in **Figure 5A**. The genes clustered into three patterns. Cluster 1 contained two genes: acetyl-CoA acetyltransferase 2 (Acat2) and 3-hydroxybutyrate dehydrogenase type 2 (Bdh2) whose expression peaked in adulthood. Cluster 2 contained four genes: 3-hydroxybutyrate dehydrogenase type 1 (Bdh1), acetyl-CoA acetyltransferase 1 (Acat1), 3-hydroxy-3-methylglutaryl-CoA synthase 2 (Hmgcs2), and 3-hydroxymethyl-3-methylglutaryl-CoA lyase (Hmgcl) whose expressions were highest during the suckling period of development. Cluster 3 contained one gene 3-oxoacid CoA transferase 1 (Oxct1) whose expression was highest during the fetal period. The FPKM values for genes involved in ketogenesis from cluster 1 and 2 of the heatmap are displayed in **Figure 5B**.

### Expression of genes involved in cholesterol and bile acid metabolism at different life stages

**Figure 6:**
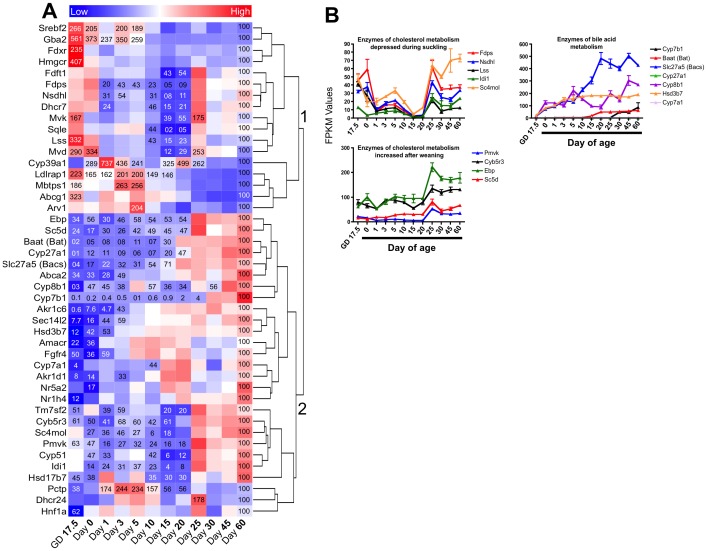
Ontogeny of cholesterol and bile acid metabolism gene expressions. A) One-way hierarchical cluster map of cholesterol and bile acid metabolism genes that changed significantly in at least one age compared to Day 60. mRNA expression of genes that were statistically different from those in mice 60 days of age (control) have the percent indicated within the heatmap square (control = 100%). B) Examples of FPKM (fragments per kilobase of exon per million reads mapped) values of individual genes of cholesterol and bile acid metabolism throughout development. For gene function information see Table S2.

To evaluate the expression of individual genes of cholesterol and bile acid metabolism, a gene list was compiled using the following gene sets: GO:0008203 (Cholesterol Metabolic Process) and GO:0008206 (Bile Acid Metabolism). The resulting gene list contained 122 genes, of which the expression of 85 genes significantly changed in at least one age compared to Day 60. A one-way hierarchical cluster map of these 85 genes is displayed in **Figure 6A**. The 85 genes clustered into two large clusters: cluster 1 contained genes that were mainly suppressed during the suckling period, but more highly expressed during the fetal stage and after weaning; and cluster 2 contained genes whose expressions were low during the fetal and suckling periods, but increased after weaning and into adulthood. **Figure 6B** depicts FPKM values at different ages for genes encoding cholesterol and bile acid metabolism enzymes. Genes encoding bile acid metabolism enzymes were prominent in cluster 2. In general, these genes showed an increase in expressions around the weaning age. The exception to this was Hsd3b7, whose expression was low during the neonatal age, but then increased 3 days after birth (**Figure 6B** **top right panel**). Genes encoding cholesterol metabolism enzymes were found in both clusters. The expression of a number of genes encoding cholesterol metabolism enzymes were depressed during the suckling period including farnesyl diphosphate synthase (Fdps), NAD(P) dependent steroid dehydrogenaselike (Nsdhl), lanosterol synthase (Lss), isopentenyl-diphosphate delta isomerase 1 (Idi1), and methylsterol monooxygenase 1 (Sc4mol) (**Figure 6B** **top left panel**). However, there were also a number of genes whose expressions increased after weaning including phosphomevalonate kinase (Pmvk), cytochrome b5 reductase 3 (Cyb5r3), emopamil binding protein (Ebp), and sterol-C5-desaturase-like (Sc5d) (**Figure 6B** **bottom left panel**).

### Expression of genes involved in glucose and glycogen metabolism at different life stages

**Figure 7:**
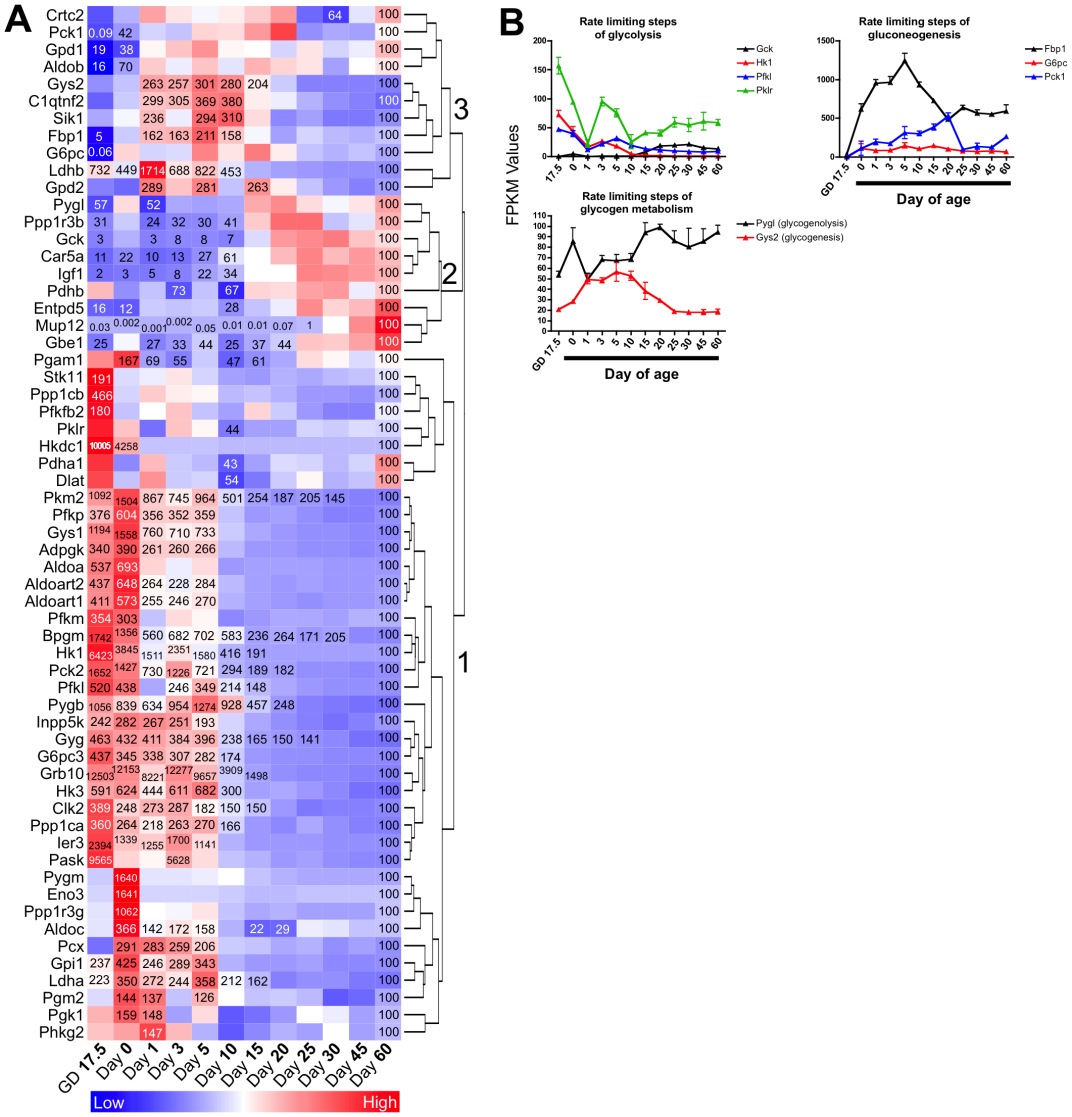
Ontogeny of glucose and glycogen metabolism gene expressions. A) One-way hierarchical cluster map of glucose and glycogen metabolism genes that changed significantly in at least one age compared to Day 60. mRNA expression of genes that were statistically different from those in mice 60 days of age (control) have the percent indicated within the heatmap square (control = 100%). B) Examples of FPKM (fragments per kilobase of exon per million reads mapped) values of individual genes of glucose and glycogen metabolism throughout development. For gene function information see Table S3.

To evaluate the expression of genes involved in glucose and glycogen metabolism, a gene list was compiled using the following gene sets: GO:0006094 (Gluconeogenesis), GO:0006096 (Glycolysis), GO:0005980 (Glycogenolysis), and GO:0005978 (Glycogenesis). The resulting list contained 92 genes of which 86 significantly changed in at least one age compared to Day 60. A one-way hierarchical cluster map of these 86 genes is displayed in **Figure 7A**. The majority of the genes were most highly expressed during the neonatal and suckling period (**Figure 7A**). Three major clusters of genes were observed. Cluster 1 contained genes that were most highly expressed only during the suckling period, whereas cluster 2 contained genes whose expressions increased after weaning. **Figure 7B** depicts FPKM values for genes encoding the rate limiting enzymes of glucose and glycogen metabolism at different ages. Genes encoding rate limiting glycolytic factors [glucokinase (Gck), hexokinase 1 (Hk1), phosphofructokinase liver (Pfkl), pyruvate kinase liver and RBC (Pklr)] were all most highly expressed at the fetal age (**Figure 7B** **top left panel**). In contrast, genes encoding rate limiting gluconeogenesis enzymes [fructose-1,6-bisphosphatase 1 (Fbp1), glucose-6-phosphatase catalytic subunit (G6pc), phosphoenolpyruvate carboxykinase 1 (Pck1)] were most highly expressed during the suckling ages. Interestingly, expression of the gene encoding the rate limiting enzyme of glycogenolysis (phosphorylase glycogen liver; Pygl) exhibited slightly reduced expression during the suckling ages; whereas expression of the gene encoding the rate limiting step of glycogenesis (glycogen synthase 2; Gys2) was most highly expressed during the suckling ages (**Figure 7B** **bottom panel**).

### Alternative splicing isoforms of genes involved in energy metabolism at different life stages

**Figure 8:**
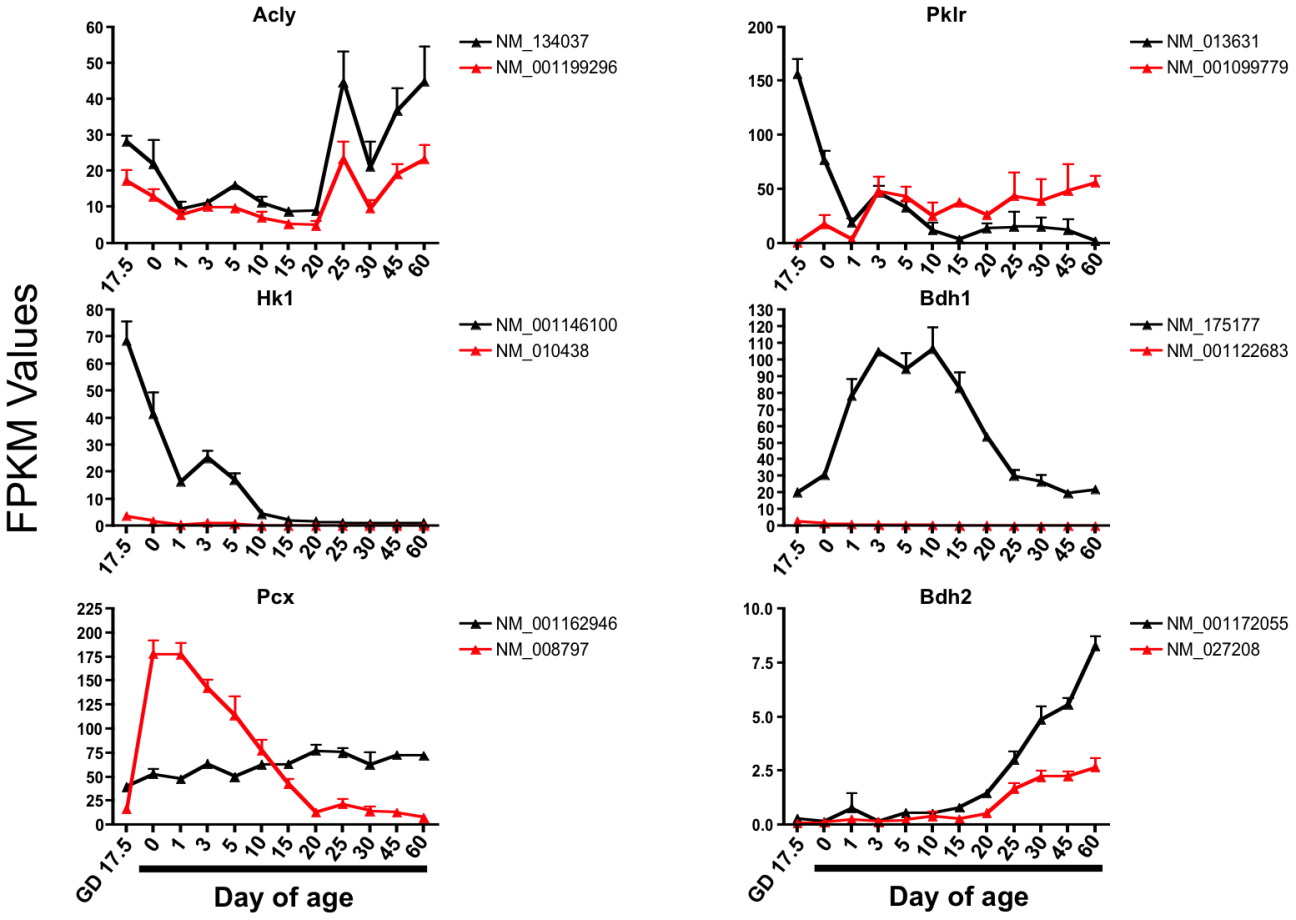
Ontogeny of the mRNA expression of known isoforms of critical genes involved in energy metabolism.

Of the 627 genes that changed significantly in at least one age compared to Day 60, 114 have alternative splicing isoforms. **Figure 8** displays the FPKM values of the alternative splicing isoforms of critical genes involved in energy metabolism including ATP citrate lyase (Acly), Pklr, Hk1, Bdh1, pyruvate carboxylase (Pcx), and Bdh2. Both isoforms of Acly showed reduced gene expression during the suckling ages. The two isoforms of Pklr showed opposing expression patterns with NM_013631 being highly expressed during the neonatal period, but then decreasing after Day 0; whereas, NM_001099779 was lowly expressed during the neonatal age then increased after Day 1. Isoforms of Hk1 (NM_010438) and Bdh1 (NM_001122683) were both lowly expressed at all ages. The expression of one isoform of Pcx (NM_001162946) was relatively constant throughout development; whereas the other isoform of Pcx (NM_008797) was highly expressed after birth and then gradually decreased in expression during the suckling period. Both isoforms of Bdh2 showed an increase in expression after weaning.

## Discussion

During liver development, gene expression profiles change over time to adapt to the changing functions of the liver [7], [8]. Elucidating molecular regulations in this developmental process is important for understanding liver functions and also useful for exploring liver diseases. High-throughput gene expression profiling techniques such as microarrays and RNA-Sequencing are well suited to reveal global developmental changes in gene expression. Our study and others [7], [9] have identified global gene expression patterns along the developmental time line of mouse liver. Using microarray analysis, Li et al., (2009) also found that a large set of genes are “turned on” at birth including gene sets involved in lipid metabolic process, fatty acid metabolic process, lipid transport, and cholesterol metabolic process. Accurate comparison of the expression of individual genes between studies, however, is difficult because the published data does not exactly match the developmental stages chosen for our study.

The diet plays a major role in regulating the expression of energy metabolism genes in the liver during development and during adulthood. Our study and others demonstrate a clear relationship between changes in the expressions of energy metabolism genes and nutrient transitions from *in utero* to suckling to weaning [10], [11], [12]. Furthermore, we have recently shown that multiple factors in the diet can affect many genes of energy metabolism in the adult liver [13]. Thus, transitions in nutrient intake throughout different life stages present an opportunity to study the effect of nutritional changes on the expression of energy metabolism genes during growth and development. This study is the first to provide a comprehensive quantification of the expression of genes implicated in energy metabolism in mice throughout ontogeny.

We and others demonstrate that, with the onset of suckling, ketogenic and lipid oxidation pathways are induced [10], [11], [12]; but how this induction is regulated is still unclear. PGC-1α (Ppargc1a) and Pparα are transcription factors known to regulate these pathways in adults [14], [15], [16]; however, Yubero *et al.* (2004) found that changes in PGC-1α expression did not parallel hepatic lipid oxidation gene induction following initiation of suckling [12]. We also did not observe a significant induction of PGC-1α expression after birth (data not shown) suggesting that this factor may not regulate lipid oxidation or ketogenic pathways during development in mice. Of the transcription factors we evaluated, only Pparα and Sirt3 were markedly induced at the onset of suckling. In rat liver, Pparα mRNA and protein increase after birth and remains enhanced during suckling [10]. Our results show a similar pattern of Pparα mRNA expression in mouse liver with highest levels observed during the suckling ages. Thus, Pparα is a good candidate factor for the regulation of ketogenic and lipid oxidation pathways that are induced at the onset of suckling. Sirt3 may also play a role in this induction as this factor is known to activate enzymes of fatty acid oxidation and ketogenesis [17], [18], and is implicated in the metabolic syndrome [19], [20]. Research into whether Sirt3 regulate ketogenesis, or lipid oxidation at birth is warranted.

We are the first to describe an in-depth analysis of the ontogeny of lipid droplet and VLDL metabolism genes. Collectively, these pathways were upregulated after birth – likely to accommodate the large influx of lipids from milk. Lipid droplets are coated with proteins, such as perilipins (encoded by Plin family genes), that are thought to regulate lipid droplet turnover by regulating lipolysis. Furthermore, lipid droplets and their associated proteins play a role in the development of insulin resistance, obesity, and the metabolic syndrome [21], [22], [23]. In this study, we observed a brief, but stark increase in Plin2 expression coincident with the onset of suckling. Interestingly, Plin2-null mice are protected from diet-induced obesity and fatty liver disease, indicating a critical role for Plin2 in the development of metabolic diseases [24]. Thus, identification of pathways that control expression of *Plin2* may provide new therapeutic targets for the treatment of obesity and related disorders. Our study presents an interesting model to investigate how changes in nutrition can regulate Plin2 in liver.

Another interesting finding from this study is that the rate limiting steps of fatty acid β-oxidation, Acox1 (peroxisomal), and Cpt1a (mitochondrial) are divergently expressed from weaning into adulthood. This may indicate an increased need for peroxisomal over mitochondrial β-oxidation after weaning. Acox1 mRNA expression increased steadily after birth, and this pattern has been shown at the protein level [25]. The pattern of Cpt1a expression that was observed in this study, induced at birth then markedly decreased at weaning, parallels Cpt1a expression profiles in rats at the mRNA, protein, and activity levels [26], [27], [28]. Pparα regulates the expression of Acox1 in adult mice [29]; however, the regulation of Cpt1a in mice is less explored. In rats, Cpt1a can be induced by administration of Clofibrate (Pparα inducer) and long chain fatty acids through independent mechanisms (reviewed in [28]). Regardless, because modulating fatty acid oxidation is an attractive therapeutic target for the treatment of obesity, further research into the mechanisms governing the divergent expression of Acox1 and Ctp1a in mice at the suckling-weaning transition are warranted.

Elovl3 regulates the elongation of saturated and mono-unsaturated fatty acids. Elovl3-null mice are resistant to diet-induced obesity [30]. We observed that, after weaning, Elovl3 mRNA expression was starkly induced; however what regulates this increase is unknown. *Elovl3* expression is induced in brown fat of cold-stimulated mice by the Ppar transcription factors [31], [32], and in liver of normal mice by Srebp1 transcription factors [33]. However, the mRNA expressions of Ppar and Srebp1 factors were not induced at weaning, suggesting that other factors may be involved in the regulation of Elovl3 expression at weaning. Further research is required to indentify these factors and may lead to novel targets for the treatment of diet-induced obesity.

Stearoyl-CoA desaturases (Scd) are rate limiting enzymes in the hepatic biosynthesis of monounsaturated fatty acids from saturated fatty acids. Studies using Scd1 gene deficient mice have revealed a beneficial effect of Scd1 inhibition in diet-induced obesity, hepatic steatosis, and insulin resistance. Thus, Scd1 is an attractive target for the treatment of many metabolic disorders [34]. In the present study, we observed a sharp upregulation of Scd1 mRNA and protein immediately after weaning, corroborating earlier reports [35]. However, which factors regulate Scd1 during weaning is unknown. Scd1 expression in adult cells is regulated by many transcription factors including Srebp1c, Pparα, C/EBP-α, Pgc1-α, and LXRα [36], [37]. From our data, LXRα is the only one of the above mentioned transcription factors whose mRNA expression was highest after weaning. These data suggest that LXRα may potentially regulate Scd1 expression at weaning; however, further research is needed to confirm this.

The mRNA expressions of many enzymes of cholesterol biosynthesis followed two major patterns of expression: either decreased expression during the suckling period (such as Fdps, Nsdhl, Lss, Idi1, and Sc4mol), which is similar to hepatic cholesterol metabolism observed in sheep [38]; or induced expression at weaning (such as Pmvk, Cyb5r3, Ebp, Sc5d). The fact that many genes followed the same pattern of expression suggests a common mechanism of regulation. Srebp2 along with its regulatory Insig proteins is known to regulate the transcription of cholesterol metabolism genes in adults [39]. Srebp2 mRNA expression did not follow either of the above mentioned gene expression patterns, suggesting other factors may be controlling cholesterol metabolism gene expression during development. Determining these factors may provide novel therapeutic targets for the control of cholesterol disorders.

RNA-sequencing provides an unbiased detection of transcripts. Thus, we were able to quantitatively compare the transcript abundances of known isoforms of genes throughout liver development. Interestingly, the alternative splicing isoforms of both pyruvate carboxylase (Pcx) and pyruvate kinase, liver (Pklr) showed age dependent switches in isoform expression dominance. We are the first to reveal that isoform 2 of Pklr is the dominant form in the adult ages; however, isoform 1 is more dominantly expressed during the neonatal ages. Additionally, isoform 2 of Pcx is only dominantly expressed over isoform 1 during the early suckling ages. These results beg the interesting question of whether these isoforms perform different functions to meet the needs of the dynamically developing liver. Unfortunately, it is currently unknown if these isoform changes impact gene function or how they might influence energy metabolism. Further research into this interesting question will help shed light on the dynamics of energy metabolism during development.

Energy metabolism fluctuates to compensate for activity level of the organism. Thus, it should be noted that this report quantified energy metabolism gene expression from mice who lead a relatively sedentary life. Whether activity level affects the developmental pattern of energy metabolism gene expression is not known.

In conclusion, this study provides a comprehensive analysis of the expression of genes encoding factors involved in energy metabolism during mouse liver development. These data shed new light on the ontogeny of homeostatic regulation of hepatic energy metabolism. Understanding the regulation of key genes involved in energy metabolism is essential to decipher the pathogenesis of metabolic diseases. By providing a large database of gene expression changes throughout development, our study provides new insights into the dynamic regulation of energy metabolism. Using development as a tool, a better understanding of the regulation of energy metabolism genes can be attained, which may ultimately provide novel therapeutic targets to better manage or treat health conditions linked with aberrant regulation of metabolic pathways.

## Supporting Information

Table S1 **Lipid metabolism genes functions.**
(PDF)

Table S2 **Cholesterol and bile acid metabolism genes functions.**
(PDF)

Table S3 **Glucose and glycogen metabolism genes functions.**
(PDF)
