# Supplementary material for: Ontogeny of Hepatic Energy Metabolism Genes in Mice as Revealed by RNA-Sequencing
Source: PLoS One. 2014 Aug 7;9(8):e104560. doi: 10.1371/journal.pone.0104560 (PMC4125194; doi:10.1371/journal.pone.0104560)
Supplement: Table S1 — Lipid metabolism genes functions. (PDF) [file pone.0104560.s001.pdf]

Table S1. Lipid metabolism genes functions.

Gene Symbol	Gene Name	Function
**Apolipoprtoteins**		
Apoa1	apolipoprotein A-I	The major protein component of HDL in plasma.
Apoa1bp	apolipoprotein A-I binding protein	Interacts with Apoa1.
Apoa2	apolipoprotein A-II	May stabilize HDL structure by its association with lipids. Therefore affects HDL metabolism.
Apoa4	apolipoprotein A4	Possible role in VLDL secretion and catabolism.
Apoa5	apolipoprotein A-V	Important determinant of plasma triglyceride levels by both being a potent stimulator of Apoc2 lipoprotein lipase and an inhibitor of hepatic VLDL-triglyceride production rate.
Apoc1	apolipoprotein C-I	Modulates the interaction of Apoe with VLDL and inhibits binding of VLDL to the LDL receptor-related protein.
Apoc2	apolipoprotein C2	VLDL component, activates lipoprotein lipase.
Apoc3	apolipoprotein C-III	Inhibits lipases and decreases the uptake of lymph chylomicrons by hepatic cells.
Apoc4	apolipoprotein C-IV	May participate in lipoprotein metabolism.
Apoe	apolipoprotein E	Mediates the binding, internalization, and catabolism of lipoprotein particles.
Apof	apolipoprotein F	Associates with LDL. Inhibits cholesteryl ester transfer protein and regulates cholesterol transport.
**Fatty acid desaturases**		
Degs1	delta(4)-desaturase, sphingolipid 1	Fatty acid desaturase. Responsible for inserting double bonds into specific positions in fatty acids
Fads1; 2; 3; 6	Fatty acid desaturase 1; 2; 3; 6	Desaturates fatty acids.
Scd1; 2	Stearoyl-CoA desaturase 1; 2	Desaturates fatty acyl-CoA substrates.
**Fatty acid elongation**		
Elovl1; 2; 3; 5; 7	ELOVL fatty acid elongase 1; 2; 3; 5; 7	Fatty acid chain elongation.
**Fatty acid synthesis**		
Acaca	acetyl-CoA carboxylase α	Rate limiting step of fatty acid synthesis. Produces malonyl-CoA from acetyl-CoA.
Acacb	acetyl-CoA carboxylase beta	Produces malonyl-CoA from acetyl-CoA and is thought to control fatty acid oxidation because malonyl-CoA inhibits Cpt1, the rate-limiting step of mitochondrial β-oxidation.
Acly	ATP citrate lyase	Synthesizes cytosolic acetyl-CoA.
Fasn	fatty acid synthase	Synthesizes long-chain saturated fatty acids.
Mcat	malonyl CoA:ACP acyltransferase	Transfers malonyl from malonyl-CoA to carriers. May have a role in mitochondrial fatty acid synthesis.
Mecr	mitochondrial trans-2-enoyl-CoA reductase	Reduces trans-2-enoyl-CoA to acyl-CoA. May have a role in mitochondrial fatty acid synthesis.
Pecr	peroxisomal trans-2-enoyl-CoA reductase	Participates in chain elongation of fatty acids.
Ptpla; Ptplad1; Ptplb	protein tyrosine phosphatase-like, member A; domain containing 1; member b	Responsible for the dehydration step in very long-chain fatty acids (VLCFAs) synthesis
**Fatty acid synthesis - mitochondria**		
Acsf3	acyl-CoA synthetase family member 3	Catalyzes the initial reaction in intramitochondrial fatty acid synthesis.
Cbr4	carbonyl reductase 4	Localized to mitochondria. May play a role in mitochondrial fatty acid synthesis.
H2-Ke6	H2-K region expressed gene 6	Regulates concentrations of estrogens and androgens. Has a role in mitochondrial fatty acid synthesis.
**Fatty alcohol synthesis**		
Far1	fatty acyl CoA reductase 1	Catalyzes the reduction of saturated fatty acyl-CoA with chain length C16 or C18 to fatty alcohols. Fatty alcohols have two metabolic fates in mammals: incorporation into ether lipids or incorporation into waxes.
**Fatty acid transport**		
Dbi	diazepam binding inhibitor	Binds medium- and long-chain acyl-CoA esters. May function as an intracellular carrier of acyl-CoA esters.
Fabp1; 2; 4; 5	fatty acid binding protein 1, liver; 2, intestinal; 4, adipocyte; ;5, epidermal	Fatty acid uptake, transport, and metabolism. Fabp1 also binds bile acids. Fabp5: polymorphisms associated with type-II diabetes.
Npc1; 2	Niemann-Pick disease, type C1; C2	Intracellular cholesterol trafficking.
Slc27a4	solute carrier family 27, member 4	Involved in translocation of long-chain fatty acids across the plasma membrane.
**Lipid droplet metabolism**		
fitm1	fat storage-inducing transmembrane protein 1 ; 2	Play important roles in lipid droplet accumulation.
Plin2	perilipin 2	Coats cellular lipid droplets. Serves as a marker of lipid accumulation.
Plin5	perilipin 5	Binds to lipid droplets and protects them from lipolytic degradation.
**Lipoprotein absorption and metabolism**		
Lcat	lecithin-cholesterol acyltransferase	Esterifies extracellular cholesterol.
Ldlr	low density lipoprotein receptor	Binds LDL, initiates endocytosis.
Lipc	lipase, hepatic	Hydrolyzes phospholipids, mono-, di-, & triglycerides.
Lmf1	lipase maturation factor 1	Resides in the endoplasmic reticulum and is involved in the maturation and transport of lipoprotein lipase.
Lpl	lipoprotein lipase	Vascular triglyceride lipases.
Lrp1; 10; 3; 5	low density lipoprotein receptor-related protein 1; 10; 3; 5	1: Involved in plasma clearance of chylomicron remnants; 10: Involved in the uptake of Apoe containing lipoproteins in liver; 3: Its precise role is unclear because it does not bind to very low density lipoprotein (VLDL) or to LRPAP1 in vitro; 5: binds and internalizes ligands in receptor-mediated endocytosis.
Mgll	monoglyceride lipase	Converts monoglycerides to fatty acids and glycerol.
Mogat2	monoacylglycerol O-acyltransferase 2	Catalyzes the formation of diacylglycerol from 2-monoacylglycerol and fatty acyl-CoA.
Mttp	microsomal triglyceride transfer protein	Lipoprotein assembly.
Pcsk9	proprotein convertase subtilisin/kexin 9	A serine protease that destroys Ldlr's in liver and thereby controls the level of LDL in plasma.
Pon1	paraoxonase 1	Protects lipoprotein particles from free radical oxidation.
Saa1	serum amyloid A1	Plays an important role in HDL metabolism and cholesterol homeostasis. Is an apolipoprotien of HDL.
Scarb1	scavenger receptor class B, member 1	Mediates cholesterol transfer to and from HDL.
Soat1; 2	sterol O-acyltransferase 1; 2	Forms fatty acid-cholesterol esters. A role in lipoprotein assembly and dietary cholesterol absorption.
Dgat2	diacylglycerol O-acyltransferase 2	Catalyzes the final reaction in triglyceride synthesis.

Table S1. Lipid metabolism genes functions.

Gene Symbol	Gene Name	Function
**Fatty acid oxidation**		
Cyp4a10; 4a14	cytochrome P450, family 4, subfamily a, polypeptide 10; 14	Participates in fatty acid oxidation in ER. Hydroxylates fatty acids such as laurate, myristate and palmitate.
Acsl5	acyl-CoA synthetase long-chain family member 5	Convert long-chain fatty acids to acyl-CoA esters. Localized to mitochondria and peroxisome.
**Mitochondrial β-oxidation**		
Acaa2	acetyl-CoA acyltransferase 2	Final step of mitochondrial β-oxidation
Acad11	acyl-CoA dehydrogenase family, member 11	Catalyzes initial steps of mitochondrial β-oxidation of saturated C22-CoAs.
Acadl	acyl-CoA dehydrogenase long chain	Catalyze the initial steps of mitochondrial β-oxidation of longt chain fatty acids.
Acadm	acyl-CoA dehydrogenase medium chain	Catalyze the initial steps of mitochondrial β-oxidation of medium chain fatty acids.
Acads	acyl-CoA dehydrogenase short chain	Catalyze the initial steps of mitochondrial β-oxidation of short chain fatty acids.
Acadvl	acyl-CoA dehydrogenase very long chain	Catalyze the initial steps of mitochondrial β-oxidation of very long chain fatty acids.
Acsm1; 3	acyl-CoA synthetase medium-chain family member 1; 3	Catalyzes addition of CoA onto medium chain fatty acids for mictochondrial import.
Cpt1a	carnitine palmitoyltransferase 1A (liver)	Rate limiting step of mitochondrial β-oxidation. Transports fatty acids into the mitochondria.
Decr1	2,4-dienoyl CoA reductase 1	Participates in the β-oxidation and metabolism of unsaturated fatty enoyl-CoA esters.
Echs1	enoyl CoA hydratase, short chain, 1	Functions in the second step of the mitochondrial fatty acid β-oxidation pathway.
Hadh	hydroxyacyl-CoA dehydrogenase	Enzyme involved in mitochondrial β-oxidation of medium chain fatty acids.
Hadha	hydroxyacyl-CoA dehydrogenase	Enzyme involved in mitochondrial β-oxidation of short chain fatty acids.
Hadhb	Mitochondrial trifunctional protein β subunit	Enzyme involved in mitochondrial β-oxidation of long chain fatty acids.
**Peroxisomal fatty acid oxidation**		
Hsd17b4; Acaa1a; Ehhadh; Hacl1	Hydroxysteroid dehydrogenase 7; acetyl-CoA acyltransferase 1a; enoyl-CoA, hydratase/3-hydroxyacyl CoA dehydrogenase; 2-hydroxyacyl-CoA lyase 1	Enzyme of peroxisomal β-oxidation.
Abcd2	Aldl1	Possibly imports fatty acids into peroxisome.
Acox1	acyl-CoA oxidase 1	First enzyme of peroxisomal β-oxidation. Catalyzes the desaturation of acyl-CoAs to 2-trans-enoyl-CoAs.
Acox3	acyl-CoA oxidase 3, pristanoyl	Involved in the desaturation of 2-methyl branched fatty acids in peroxisomes.
Crot	carnitine O-octanoyltransferase	Converts pristanic acid β-oxidation product into a carnitine ester. This transesterification is necessary for transport of acyl-CoA molecules out of the peroxisome to the cytosol and mitochondria.
Decr2	2,4-dienoyl CoA reductase 2,	Participates in the peroxisomal β-oxidation and metabolism of unsaturated fatty enoyl-CoA esters.
Phyh	phytanoyl-CoA 2-hydroxylase	Involved in the α-oxidation of 3-methyl branched fatty acids.
Slc25a17	solute carrier family 25, member 17	Transports free CoA, FAD and NAD(+) from cytosol into the peroxisome by a counter-exchange mechanism.
Slc27a2	solute carrier family 27, member 2	Activates precursors of bile acids before peroxisomal β-oxidation.
Tysnd1	trypsin domain containing 1	Processes the peroxisomal targeting signals of proteins involved in β-oxidation of fatty acids.
**Regulation of lipid metabolism**		
Adipor1; 2	adiponectin receptor 1; 2	Adiponectin receptor mediates Ampk, Ppara ligand activity, fatty acid oxidation and glucose uptake.
Brca1	breast cancer 1, early onset	Inhibits lipid synthesis by binding to inactive phosphorylated Acaca and preventing its dephosphorylation.
Hnf1a	HNF1 homeobox A	Transcription factor required for the expression of several liver-specific genes.
Hnf4a	hepatocyte nuclear factor 4, α	Transcriptionally controlled transcription factor. Transcribes genes such as Apoc3 and Hnf1a.
Insig1; 2	Insulin induced gene 1; 2	Inhibits processing of Srebp's.
Irs1	insulin receptor substrate 1	Phosphorylated by the insulin receptor and activates proteins such as phosphatidylinositol 3-kinase.
Irs2	insulin receptor substrate 2	Mediates intracellular effects of insulin and insulin-like growth factor 1.
Lepr	leptin receptor	Modulates triglyceride metabolism. Lepr gene deletion causes lipid accumulation and increased VLDL.
Lias	lipoic acid synthetase	Involved in the synthesis of lipoic acid (a cofactor for many enzyme complexes of intermediary metabolism).
Lipt2	lipoyl(octanoyl) transferase 2 (putative)	Enzyme of lipoic acid metabolism.
Lpin1; 2; 3	lipin 1; 2; 3	Plays important roles in controlling the metabolism of fatty acids at differents levels.
Mid1ip1	MID1 interacting protein 1	Up-regulates Acaca enzyme activity.
Mlxipl	MLX interacting protein-like	Transcription factor: activates carbohydrate response elements in triglyceride synthesis genes promoters.
Mlycd	malonyl-CoA decarboxylase	Catabolizes malonyl-CoA, which inhibits mitochondrial fatty acid transport.
Mtor	mechanistic target of rapamycin	Regulates lipid synthesis via the Srebf1 pathway and mitochondrial biogenesis via Ppargc1a.
Nr1h2 (LXRβ)	nuclear receptor subfamily 1, group H, member 2	Nuclear receptor which is a regulator of lipid homeostasis.
Nr1h3	nuclear receptor subfamily 1, group H, member 3	Plays an important role in the regulation of cholesterol homeostasis, regulating cholesterol uptake through MYLIP-dependent ubiquitination of LDLR, VLDLR and LRP8.
Oxsm	3-oxoacyl-ACP synthase, mitochondrial	Synthesizes lipoic acid as well as longer chain fatty acids required for optimal mitochondrial function.
Ppard	peroxisome proliferator-activated receptor delta	Regulates the peroxisomal β-oxidation pathway of fatty acids.
Pparg	peroxisome proliferator-activated receptor gamma	Once activated by a ligand, this receptor binds to a promoter element in the gene for acyl-CoA oxidase and activates its transcription. It therefore controls the peroxisomal β-oxidation pathway of fatty acids.
Prkaa1; Prkaa2; Prkab1; Prkag1	protein kinase, AMP-activated, α 1 catalytic subunit; α 2 catalytic subunit; β 1 non-catalytic subunit; γ 1 non-catalytic subunit	Subunits of Ampk – in response to ATP levels, activates energy-producing, and inhibits energy-consuming processes.
Srebf1	Sterol regulatory element binding transcription factor 1	Regulates transcription of fatty acid synthesis genes.
Trib3	tribbles homolog 3	Disrupts insulin signaling. Inhibits Akt kinases.
Acot2	acyl-CoA thioesterase 2	Catalyzes acyl-CoAs to free fatty acid and CoA, thus regulating levels of acyl-CoAs, free fatty acids and CoA.
