# Supplementary material for: Ontogeny of Hepatic Energy Metabolism Genes in Mice as Revealed by RNA-Sequencing
Source: PLoS One. 2014 Aug 7;9(8):e104560. doi: 10.1371/journal.pone.0104560 (PMC4125194; doi:10.1371/journal.pone.0104560)
Supplement: Table S2 — Cholesterol and bile acid metabolism genes functions. (PDF) [file pone.0104560.s002.pdf]

Table S2. Cholesterol and bile acid metabolism genes functions.

Gene Symbol	Gene Name	Function
**Regulation of cholesterol and/or bile acid metabolism**		
Fgfr4	fibroblast growth factor receptor 4	Receptor for Fgf15. Decreases Cyp7a1 expression, the rate-limiting enzyme in bile acid (BA) synthesis.
Nr1h4 (FXR)	nuclear receptor subfamily 1, group H, member 4	Receptor for BAs. Regulates expression of BAsynthesis and transport genes.
**Arv1**	ARV1 homolog (S. cerevisiae)	Critical role in sterol movement from the ER and in the regulation of cholesterol and BA metabolism.
Hnf1a	HNF1 homeobox A	Hnf1a-null mice have defective BA transport and HDL metabolism, increased BA and cholesterol synthesis.
Nr5a2 (Lrh1)	nuclear receptor subfamily 5, group A, member 2	Key regulator of Cyp7a expression in liver. Linked to a variety of processes, such as bile acid metabolism and reverse cholesterol transport.
**Mbtps1**	membrane-bound transcription factor peptidase, site 1	Catalyzes the first step in the proteolytic activation of the Srebf proteins.
**Srebf2**	sterol regulatory element binding transcription factor 2	Transcription factor that controls cholesterol homeostasis by transcribing sterol-regulated genes.

Gene Symbol	Gene Name	Function
**Bile acid metabolism**		
Akr1c6	aldo-keto reductase family 1, member C1	May have a role in the transport and intrahepatic concentration of bile acids.
Akr1d1	aldo-keto reductase family 1, member D1	Reduces BA intermediates 7-α,12-α-dihydroxy-4-cholesten-3-one & 7-α-hydroxy-4-cholesten-3-one.
Amacr	alpha-methylacyl-CoA racemase	Responsible for the conversion of pristanoyl-CoA and C27-bile acyl-CoAs to their (S)-stereoisomers.
Baat (Bat)	bile acid CoA: amino acid N-acyltransferase	Conjugates C24 bile acids to glycine or taurine before excretion into bile canaliculi.
Cyp27a1	cytochrome P450, family 27, subfamily A, polypeptide 1	Oxidizes cholesterol intermediates as part of the bile synthesis pathway.
Cyp39a1	cytochrome P450, family 39, subfamily A, polypeptide 1	Involved in bile acid metabolism. Has a preference for 24-hydroxycholesterol, and converts it into a 7-α-hydroxylated product.
Cyp7a1	cytochrome P450, family 7, subfamily A, polypeptide 1	Catalyzes a rate-limiting step bile acid biosynthesis by introducing a hydrophilic moiety at position 7 of cholesterol.
Cyp7b1	cytochrome P450, family 7, subfamily B, polypeptide 1	Catalyzes the first reaction in the cholesterol catabolic pathway of extrahepatic tissues, which converts cholesterol to bile acids.
Cyp8b1	cytochrome P450, family 8, subfamily B, polypeptide 1	Responsible for the balance of cholic acid and chenodeoxycholic acid formation.
Gba2	glucosidase, beta (bile acid) 2	Catalyzes the hydrolysis of bile acid 3-O-glucosides.
Hsd3b7	Hydroxy-δ-5-steroid dehydrogenase, 3 β and steroid δ-isomerase 7	Enzyme of bile acid synthesis.
Slc27a5 (Bacs)	solute carrier family 27, member 5	Activates C24 BAs to their CoA thioesters. First step in conjugating bile acids to glycine or taurine.

Gene Symbol	Gene Name	Function
**Cholesterol metabolism**		
Abca2	ATP-binding cassette, sub-family A (ABC1), member 2	Plays a role in the trafficking of LDL-derived free cholesterol.
Abcg1	ATP-binding cassette, sub-family G (WHITE), member 1	Macrophage cholesterol efflux.
Cyb5r3	cytochrome b5 reductase 3	Enzyme of cholesterol biosynthesis.
Cyp51	Cyp51 cytochrome P450, family 51	Catalyzes the removal of the 14α-methyl group from lanosterol during cholesterol synthesis.
Dhcr24	24-dehydrocholesterol reductase	Reduces the δ-24 double bond of sterol intermediates during cholesterol synthesis.
Dhcr7	7-dehydrocholesterol reductase	Catalyzes the final step of cholesterol synthesis.
Ebp	emopamil binding protein	Catalyzes the conversion of δ(8)-sterols to their δ(7)-isomers during cholesterol synthesis.
Fdft1	farnesyl-diphosphate farnesyltransferase 1	First specific enzyme in cholesterol synthesis. Converts farnesyl diphosphate to squalene.
Fdps	farnesyl diphosphate synthase	Enzyme in cholesterol synthesis.
Fdxr	ferredoxin reductase	Hydroxylates sterol C-27 in cholesterol metabolism.
Hmgcr	3-hydroxy-3-methylglutaryl-CoA reductase	Rate-limiting enzyme in cholesterol synthesis.
Hsd17b7	hydroxysteroid (17-beta) dehydrogenase 7	Enzyme of cholesterol synthesis.
Idi1	isopentenyl-diphosphate delta isomerase 1	Catalyzes the formation of the substrates for the synthesis of farnesyl diphosphate.
Lss	lanosterol synthase	Enzyme of cholesterol synthesis.
Mvd	mevalonate (diphospho) decarboxylase	Converts mevalonate pyrophosphate into isopentenyl pyrophosphate in cholesterol synthesis.
Mvk	mevalonate kinase	Enzyme of cholesterol synthesis. May be a regulatory site in cholesterol biosynthetic pathway.
Nsdhl	NAD(P) dependent steroid dehydrogenase-like	Localized in the endoplasmic reticulum and is involved in cholesterol synthesis.
Pctp	phosphatidylcholine transfer protein	Role in cholesterol esterification.
Pmvk	phosphomevalonate kinase	Conversts mevalonate 5-phosphate into mevalonate 5-diphosphate in cholesterol synthesis.
Sc4mol	methylsterol monooxygenase 1	Localized to the ER membrane and is believed to function in cholesterol synthesis.
Sc5d	sterol-C5-desaturase-like	Enzyme of cholesterol synthesis. Converts lathosterol into 7-dehydrocholesterol.
Sec14l2	SEC14-like 2 (S. cerevisiae)	Stimulates squalene monooxygenase - a downstream enzyme in cholesterol syntheses.
Sqle	squalene epoxidase	Catalyzes the first oxygenation step in sterol synthesis. Is possibly a rate-limiting step.
Tm7sf2	transmembrane 7 superfamily member 2	Involved in the conversion of lanosterol to cholesterol.
