# Supplementary material for: Ontogeny of Hepatic Energy Metabolism Genes in Mice as Revealed by RNA-Sequencing
Source: PLoS One. 2014 Aug 7;9(8):e104560. doi: 10.1371/journal.pone.0104560 (PMC4125194; doi:10.1371/journal.pone.0104560)
Supplement: Table S3 — Glucose and glycogen metabolism genes functions. (PDF) [file pone.0104560.s003.pdf]

Table S3. Glucose and glycogen metabolism genes functions.

Gene Symbol	Gene Name	Function
**Glycerol metabolism**		
Gpd1; 2	glycerol-3-phosphate dehydrogenase 1; 2	Critical role in carbohydrate and lipid metabolism. Reversibly catalyzes dihydroxyacetone phosphate (DHAP) to glycerol-3-phosphate. Reoxidize NADH formed during glycolysis. Gpd1: cytosol, Gpd2: mitochondria.
**Anearobic glycolysis**		
Ldha; Ldhb	lactate dehydrogenase A; B	Catalyzes the conversion of L-lactate and NAD to pyruvate and NADH in the final step of anaerobic glycolysis.
**Glucose metabolism (glycolysis and gluconeogenesis)**		
Adpgk	ADP-dependent glucokinase	Catalyzes the ADP-dependent phosphorylation of glucose to glucose-6-phosphate. May play a role in glycolysis during ischemic conditions.
Dlat	dihydrolipoamide S-acetyltransferase	The pyruvate dehydrogenase complex catalyzes pyruvate to acetyl-CoA - the link between glycolysis and the tricarboxylic acid cycle. Dihydrolipoamide acetyltransferase is one of the 3 enzymatic components of the complex.
Eno3	enolase 3	Enzyme of glycolysis.
Entpd5	ectonucleoside triphosphate diphosphohydrolase 5	Part of an ATP hydrolysis cycle that converts ATP to AMP resulting in a compensatory increase in aerobic glycolysis. Plays a role in the Akt1-Pten signaling pathway by promoting glycolysis in proliferating cells.
Fbp1	fructose-1,6-bisphosphatase 1	A gluconeogenesis regulatory enzyme, catalyzes the hydrolysis of fructose 1,6-bisphosphate to fructose 6-phosphate.
Gck	glucokinase (hexokinase 4)	Catalyzes glucose to glucose-6-phosphate, the first step in glycolysis. In contrast to other forms of hexokinase, this enzyme is not inhibited by its product glucose-6-phosphate but remains active while glucose is abundant.
Hk1	hexokinase 1	Catalyzes glucose to glucose-6-phosphate. Couples glycolysis to mitochondrial oxidative phosphorylation. Activated by Akt/PKB-mediated phosphorylation and inhibited by glucose-6-phosphate.
Hk3	hexokinase 3	Phosphorylates glucose to glucose-6-phosphate. Is inhibited by its product, glucose-6-phosphate.
Hkdc1	hexokinase domain containing 1	Putative hexokinase.
Pdha1	pyruvate dehydrogenase α 1	The pyruvate dehydrogenase complex catalyzes pyruvate to acetyl-CoA - the link between glycolysis and the tricarboxylic acid cycle. This gene encodes the E1 α 1 subunit of the complex, containing the active site.
Pdhb	pyruvate dehydrogenase β	The pyruvate dehydrogenase complex catalyzes pyruvate to acetyl-CoA - the link between glycolysis and the tricarboxylic acid cycle. This gene encodes the E1 beta subunit of the complex.
Pfkl; Pfkm; Pfkp	phosphofructokinase, liver; muscle; plateletl	Catalyzes a rate limiting step in glycolysis, D-fructose 6-phosphate to D-fructose 1,6-bisphosphate.
Pklr; Pkm2	pyruvate kinase, liver and RBC; muscle	Catalyzes phohsphoenolpyruvate into pyruvate and ATP, which is a rate-limiting step of glycolysis.
Car5a	carbonic anhydrase VA	May play an important role gluconeogenesis. Localized to mitochondria.
Pck1	phosphoenolpyruvate carboxykinase 1	Main rate limiting step of gluconeogenesis. Catalyzes the formation of phosphoenolpyruvate from oxaloacetate. Expression is regulated by insulin, glucocorticoids, glucagon, cAMP, and diet. Localized to cytosol.
Pck2	phosphoenolpyruvate carboxykinase 2	Mitochondrial enzyme that catalyzes the conversion of oxaloacetate to phosphoenolpyruvate in the presence of GTP. not modulated by hormones such as glucagon and insulin that regulate the cytosolic form.
Pcx	pyruvate carboxylase	Catalyzes the initial reaction of gluconeogenesis, carboxylation of pyruvate to oxaloacetate.
Aldoa; b; c	aldolase A; B; C, fructose-bisphosphate	Glycolytic enzyme that catalyzes the reversible conversion of fructose-1,6-bisphosphate to glyceraldehyde 3-phosphate and dihydroxyacetone phosphate. Aldolase A expression is repressed in adult liver.
Aldoart1; Aldoart2	aldolase 1 A retrogene 1; 2	Aldoa retrogene.
Bpgm	2,3-bisphosphoglycerate mutase	Enzyme of glycolysis and gluconeogenesis.
Gpi1	glucose-6-phosphate isomerase 1	Enzyme of glycolysis and gluconeogenesis. Catalyzes the reversible isomerization of glucose-6-phosphate and fructose-6-phosphate.
Pgam1	phosphoglycerate mutase 1	Enzyme of glycolysis and gluconeogenesis. Catalyzes the reversible reaction of 3-phosphoglycerate to 2-phosphoglycerate.
Pgk1	phosphoglycerate kinase 1	A glycolytic enzyme that catalyzes the conversion of 1,3-diphosphoglycerate to 3-phosphoglycerate.
**Gluconeogenesis and glycogenolysis**		
G6pc; G6pc3	glucose-6-phosphatase catalytic subunit; catalytic 3	Catalytic subunits of glucose-6-phosphatase. Catalyzes the hydrolysis of glucose-6-phosphate to glucose and phosphate in the last step of the gluconeogenic and glycogenolytic pathways. Hence, it is the key enzyme in homeostatic regulation of blood glucose levels.

Table S3. Glucose and glycogen metabolism genes functions.

Gene Symbol	Gene Name	Function
**Regulation of glucose and glycogen metabolism**		
C1qtnf2	C1q and tumor necrosis factor related protein 2	Adiponectin paralog. Activates Ampk and Acaca, increasing glycogenesis and fatty acid oxidation.
Grb10	growth factor receptor-bound protein 10	Suppresses signals from receptors including the insulin and insulin-like growth factor receptors.
Pask	PAS domain containing serine/threonine kinase	A sensor of energy homeostasis: regulates glycogen synthesis by phosphorylating and inhibiting Gys1.
Ppp1ca; Ppp1cb; Ppp1r3b; Ppp1r3g	protein phosphatase 1 (Pp1), catalytic subunit, α isozyme; β isozyme; regulatory subunit 3B; regulatory subunit 3G	Ppp1ca & Ppp1cb: Catalytic subunits of Pp1 (regulates glycogen metabolism). Ppp1r3b & Ppp13rg: Glycogen-targeting subunits that suppress Pp1 inactivation of glycogen phosphorylase and enhances Pp1 activation of glycogen synthase, limiting glycogenolysis.
Inpp5k	inositol polyphosphate-5-phosphatase K	Possible role in regulating glycogenesis and glyogenolysis.
Clk2	CDC-like kinase 2	Suppresses gluconeogenesis by repressing Ppargc1a transcriptional activity on gluconeogenic genes.
Crtc2	CREB regulated transcription coactivator 2	Regulates gluconeogenesis as a component of the Lkb1/Ampk/Torc2 signaling pathway.
Ier3	immediate early response 3	May regulate the balance between energy provision and ROS production under hypoxic conditions.
Igf1	insulin-like growth factor 1	Mediates pre- and postnatal growth.
Mup12	major urinary protein 12	Secreted by liver, binds to pheromones and is excreted in urine. Inhibits gluconeogenic gene expression.
Pfkfb2	6-phosphofructo-2-kinase/fructose-2,6-biphosphatase 2	Involved in metabolism of fructose-2,6-bisphosphate, a regulatory molecule of glycolysis.
Sik1	salt-inducible kinase 1	Regulator of hepatic gluconeogenesis by repressing Torc1/Crtc1 and Torc2/Crtc2, inhibiting CREB activity.
Stk11	serine/threonine kinase 11	Reguljates glucose homeostasis in liver by activating Ampk catalytic subunits Prkaa1 and Prkaa2.
**Glycogen metabolism (glycogenesis and glycogenolysis)**		
Gyg	glycogenin	Catalyzes short glucose polymers from UDP-glucose, initial step in lgycogen formation.
Gys1; 2	glycogen synthase 1 (muscle); 2 (liver)	Rate-limiting step of glycogenesis. Transfers glucose from UDP-glucose to a glycogen molecule branch.
Phkg2	phosphorylase kinase, gamma 2	Catalytic subunit of phosphorylase b kinase – mediates glycogenolysis by activating glycogen phosphorylase.
Pygb; Pygl; Pygm	phosphorylase glycogen, brain; liver; muscle	Cleaves α-1,4-glucosidic bonds to release glucose-1-phosphate from glycogen stores. First step in glycogenolysis.
Pgm2	phosphoglucomutase 2	Interconverts of glucose-1-phosphate to glucose-6-phosphate. Links glucose to glycogen metabolism.
Gbe1	glucan (1,4-alpha-), branching enzyme 1	Glycogen branching enzyme - increases glycogen solubility, thereby reducing cellular osmotic pressure.
